# Multiple giant coronary aneurysms in adult patient managed by exclusion with bypass, case report

**DOI:** 10.1186/1749-8090-8-S1-O183

**Published:** 2013-09-11

**Authors:** S Jaber, H Altaani

**Affiliations:** 1 Surgery Department, Queen Alia Heart Institute, Ammam, Jordan

## Background

Aneurysm of the coronary artery is rare disease especially in adults,the most common cause is atherosclerosis, other causes are connective tissue diseases, vacuities and trauma and it could be idiopathic. The complications are ischemia, infarction or rupture. Treatment strategies include anticoagulation, custom made covered stents, reconstruction, resection, and exclusion with bypass.

## Case report

A 48 year old male patient presented with angina on effort, chest x-ray revealed increase in the cardiac shadow, CT scan showed multiple aneurismal dilatations in all coronary arteries, with evidence of calcifications, the largest one was 5x5 cm in diameter which was in the proximal segment of left anterior descending artery extended to the proximal left main stem.

Coronary angiography showed the same results, but the distal segment of all arteries were free of dilatation, echocardiography showed normal ejection fraction.

Our management was by midsternotomy incision, partial cardiopulmonary bypass, with antegrade and retrograde cardioplegia, we bypassed the coronaries by saphenous vein graft to OM,s and PD of RCA and LIMA to distal LAD The ascending aorta was opened left main was closed from inside the aorta, the RCA was legated from its proximal segment, and just distal to the most distal aneurysm, the same done for LAD and Cx .the patient came off bypass smoothly, extubated after 4 hours and he was discharged from the hospital after 6 days with uneventfully early outcome.

## Conclusion

Multiple coronary aneurysms in adults is very rare entity, ischemia is the main presenting complication, many options for treatment depend on the size, distribution, and complications, in our patient the exclusion with bypass was good option with good results but it needs long term follow-up of the patient for long term results.

